# Higher frequency of osteoarthritis in patients with ACL graft rupture than in those with intact ACL grafts 30 years after reconstruction

**DOI:** 10.1007/s00167-019-05726-6

**Published:** 2019-10-29

**Authors:** Tomas Söderman, Marie-Louise Wretling, Mari Hänni, Christina Mikkelsen, Robert J. Johnson, Suzanne Werner, Anders Sundin, Adel Shalabi

**Affiliations:** 1 Department of Radiology, Institution of Surgical Sciences, Uppsala University, Akademiska Hospital, 751 85 Uppsala, Sweden; 2 Department of Radiology, Karolinska University Hospital, Stockholm, Sweden; 3 Department of Molecular Medicine and Surgery, Stockholm Sports Trauma Research Center, Karolinska Institutet, 171 76 Stockholm, Sweden; 4 Department of Orthopedics and Rehabilitation, University of Vermont, Burlington, VT 05405 USA

**Keywords:** ACL reconstruction, Knee laxity, Long-term evaluation, Magnetic resonance imaging, Osteoarthritis

## Abstract

**Purpose:**

The aim was to assess the results of anterior cruciate ligament (ACL) reconstruction regarding graft failure, knee laxity, and osteoarthritis (OA) from a longterm perspective. It was hypothesized that intact ACL graft reduces the risk for increased OA development.

**Methods:**

The cohort comprised 60 patients with a median follow-up 31 (range 28–33) years after ACL reconstruction. They were evaluated with magnetic resonance imaging, radiography, KT-1000 arthrometer and the pivot shift test.

**Results:**

Out of the 60 patients, 30 (50%) showed an intact ACL graft and 30 (50%) a ruptured or absent ACL graft. Patients with ruptured ACL grafts had more medial tibiofemoral compartment OA than those with an intact ACL graft (*p* = 0.0003). OA was asymmetric in patients with ruptured ACL grafts with more OA in the medial than in the lateral tibiofemoral compartment (*p* = 0.013) and the patellofemoral compartment (*p* = 0.002). The distribution of OA between compartments was similar in patients with an intact ACL graft. KT-1000 values of anterior knee laxity were higher in patients with ruptured compared to those with intact ACL grafts (*p* = 0.012). Side-to-side comparisons of anterior knee laxity showed higher KT-1000 values in patients with ruptured ACL graft (*p* = 0.0003) and similar results in those with intact graft (*p* = 0.09). The pivot shift grade was higher in the group with a ruptured ACL graft (*p* < 0.0001).

**Conclusions:**

Median 31 (range 28–33) years after ACL reconstruction, 50% of the patients showed an intact ACL graft and no side-to-side difference regarding anterior knee laxity. Patients with ruptured ACL grafts had more OA of the medial tibiofemoral compartment than those with intact ACL grafts.

**Level of evidence:**

Retrospective cohort study, Level III.

## Introduction

The anterior cruciate ligament (ACL) is commonly injured with a reported injury rate between 78 and 81 injuries per 100,000 individuals and year [31]. ACL injury results in an abnormal knee joint laxity due to an increase of anterior translation of the tibia in relation to abnormal elongation and absence of the ACL. This laxity often refers to a feeling of giving way of the knee, due to loss of function of the ACL, leading to pain and varying degrees of disability, ranging from limitations in sports participation to difficulties performing activities of daily living. An ACL injury is also often combined with meniscal tears, articular cartilage injuries and posttraumatic osteoarthritis (OA) [7, 25]. The prevalence of OA after ACL injuries varies considerably between studies (10–90%), and it has been reported that ACL reconstruction cannot prevent the development of OA [14, 24, 27, 28, 30, 32]. Although the prevalence of OA after ACL reconstruction is higher in the operated knee than in the non-injured knee [17, 18, 25, 27, 32, 34, 43], it needs to be considered that the cause of OA is multifactorial. Long-term outcome after an ACL injury is influenced mainly by the presence of associated injuries, such as those of the meniscus and articular cartilage [11, 22, 25, 28, 33].

The ACL is a primary restraint to anterior tibia movement in relation to the femur and ACL deficiency can be evaluated using KT-1000 arthrometer, other clinical tests as well as magnetic resonance imaging (MRI) and arthroscopy [3]. Achieving rotatory control of the knee after ACL reconstruction has been shown to decrease functional instability. The rotatory component can be assessed by the pivot shift test [4].

The purpose of the present investigation was to report and describe the outcome of a long-term follow-up after ACL reconstruction in terms of possible graft failure, knee laxity and OA. The hypothesis of the study was that intact ACL graft reduces the risk for increased OA development.

## Material and methods

This is a retrospective descriptive investigation based on clinical and radiological assessments 28–33 years after ACL reconstruction.

Approval for the present study was obtained from the ethics committee at the Karolinska Institutet in Stockholm, Sweden (Dnr 98/115).

### Patients

This cohort includes 134 patients who underwent ACL reconstruction using a patellar tendon bone-to-bone graft at the Karolinska University Hospital between 1968 and 1973. In the first follow-up in 1978, a total of 87 patients were included [20]. At that time, Karolinska University Hospital was the only hospital in Sweden where ACL reconstructions were performed. Therefore, patients from the entire country were referred to the Karolinska University Hospital for ACL reconstruction.

The study cohort was somewhat heterogeneous in the sense that some of the patients at the time of the index trauma already had experienced previous injuries to the knee (some requiring surgery) and were entering the study diagnosed with a collateral ligament injury, meniscal tears and/or OA. Exact knowledge of the lesions within the knee, besides damage to the ACL, was difficult to assess with certainty. Furthermore, all patients had initially been treated non-operatively for the ACL rupture. However, all patients of this cohort were later referred to surgery because of knee joint instability that prevented them from returning to their desired physical activities. The majority of the ACL injuries were sports-related and the diagnosis was confirmed by clinical tests, such as the Lachman test and the pivot shift test in combination with radiological imaging, which at the time comprised arthrography that utilized plain film examination following injection of an iodine-based contrast medium into the knee joint. Consecutive patients who underwent ACL reconstruction from January 1968 to December 1973 were included in the present investigation. Patients who underwent the procedure 1966–1967 were not included in order to avoid the early phases of the surgeons “learning curve” and the risk of initial technical errors that may have influenced the study results.

**Fig. 1:**
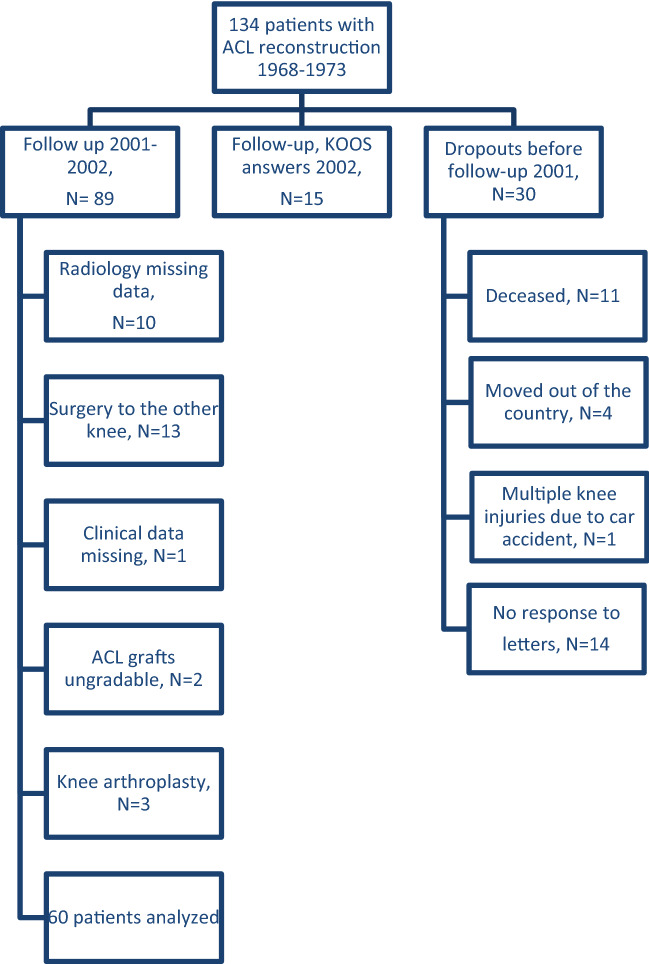
Flowchart of the patients included in the present follow-up study mean 31 years after ACL reconstruction. This figure also shows the number of dropouts of the original 134 patients before the start of the present study. These are presented to the right. Fifteen patients were included in the present study, but they were not able to come to Stockholm for the clinical examination, they only finished the KOOS (middle), which will be reported elsewhere. The patients analyzed in the present follow-up are presented to the left. *ACL *anterior cruciate ligament, *KOOS *Knee injury Osteoarthritis Outcome Score

At the time of median 31-year follow-up (range 28–33), 11 patients were deceased and four patients had moved abroad. The remaining 119 out of the initially included 134 patients were contacted by letter and asked for participation in the present study performed 2001/2002. Eighty-nine out of these 119 patients were evaluated at the Karolinska University Hospital. Because of missing radiology data (*n* = 10), surgery to the other knee (*n* = 13), missing clinical data (*n* = 1), ungradable ACL grafts (*n* = 2) and patients with knee prosthesis (*n* = 3) 29 patients were excluded and 60 patients remained for the present assessment (Fig. 1).

There were 55 men (92%) and 5 women (8%) with a median age 26 (range 17–47) years at index surgery. Fifty-eight percent of the patients injured their right knee and 42% their left knee. The median follow-up period was 31 (range 28–33) years. Median age at follow-up was 57 (range 45–79) years.

### Surgical technique

The patellar tendon reconstruction of the ACL was performed according to a standardized method at the Karolinska University Hospital developed 1966 by Broström and Eriksson, two out of seven orthopedic surgeons that performed the surgical procedures [9, 13]. The medial one-third of the patellar tendon was used as a graft. The graft was not detached distally and a small bone block was removed from the attachment site of the graft to the patella. The graft was then passed through a tibial tunnel to the attachment site on the distal femur. A small femoral tunnel was made and sutures coapted the bone block within the proximal end of the graft to the attachment site of the ACL in the notch. Associated surgery performed at the same time as index surgery included meniscus resection and repair of the medial collateral ligament. The method used was published by Eriksson 1976 [13].

### Radiological examinations

In the present follow-up 2001/2002 all patients underwent radiographic and MRI examinations of the index knee. Weight-bearing anteroposterior view, lateral and a skyline view of the patellofemoral compartment were acquired [1, 10]. The MRI examination was performed on a low field, 0.2 T scanner (Esaote, Arthroscan) to obtain sagittal (T2 STIR 5 mm, T2 5 mm, T1 5 mm, T1 4 mm, and T1 3 mm) and coronal (T2 STIR 5 mm and T1 5 mm) images*.* The imaging examinations were assessed in consensus by two experts in musculoskeletal radiology with more than 20 years’ experience. The radiographs were used to find signs of OA and the position of the tibial graft tunnel and MRI to assess the menisci and the structural integrity of the ACL graft.

### MRI assessment

The ACL graft was graded as intact (1), ruptured or missing (2) or impossible to evaluate due to artifacts (3). The menisci were classified as 1 (normal), 2 (small/defect), 3 (rupture) or 4 (missing).

### Radiographic assessment

The Kellgren–Lawrence (KL) classification [23, 39] was used to determine the degree of osteoarthritis. The radiological findings were classified as: 0 = no changes, grade 1 = doubtful narrowing of joint space and possible osteophytic lipping, grade 2 = osteophytes and possible narrowing of joint space, grade 3 = moderate multiple osteophytes, narrowing of joint space and some sclerosis and possible deformity of bone ends, grade 4 = large osteophytes, marked narrowing of joint space, severe sclerosis and deformity of bone ends.

The tibial graft tunnel was assessed in the sagittal plane. Where the tunnel emerged on tibia was graded as 1 = anterior third of the tibia in the sagittal plane, 2 = middle third of the tibia in the sagittal plane, 3 = rear third of the tibia in the sagittal plane.

### Knee joint laxity assessment

ACL graft laxity assessment was performed by using the KT-1000 arthrometer (MED metric^®^ Corp, San Diego, CA, USA). The KT-1000 measures anterior–posterior translation in millimeters (mm). The reliability and reproducibility [35, 44], as well as the sensitivity and specificity of the KT-1000, have previously been analyzed and evaluated [2, 26, 42]. The KT-1000 assessment assumes a normal contralateral ACL. A physiotherapist performed all KT-1000 measurements with an intrarater reliability of *r* = 0.984 (left knee) and *r* = 0.949 (right knee) and with > 10 years of experience using the KT-1000. A side-to-side difference of 3 mm or more was defined as a mechanical laxity and an indication of abnormal knee laxity [12, 35, 42]. To assess anterior translation of the tibia with the KT-1000, the patient’s knees were placed in 20°–30° of flexion with symmetrical tibia rotation maintained by a foot-rest. Anterior displacement of the tibia was measured using manual maximum. Both knees were measured and the data were reported in mm as well as the difference between involved and non-involved knees.

Abnormal knee joint laxity was evaluated by clinical examination using the pivot shift test. It was performed by a surgeon that had not been involved in the previous ACL surgery. The pivot shift test was graded as normal, nearly normal when a glide was present, abnormal when a clunk was found, and severely abnormal if the knee demonstrated gross shifting according to criteria established by the International Knee Documentation Committee (IKDC) [16].

### Statistical analysis

All data were presumed to be nonparametric. A Mann–Whitney *U* test was used for group comparisons in terms of intact and ruptured ACL grafts. Spearman’s test was used for correlation analysis. Statistical significance was set at a *p* value of < 0.05. No mathematical correction was made for multiple comparisons [37].

## Results

### MRI assessment

Out of the 60 patients, 30 (50%) had an intact ACL graft and 30 (50%) showed either an ACL graft rupture or a missing ACL graft.

Before or in connection with the index surgery 15 (25%) out of 60 patients had been treated with meniscus resection of the medial meniscus and four (7%) of the lateral meniscus. The distribution of meniscus injuries is shown in Table 1.

**Table 1:** Distribution of meniscal injuries (*N* = 30)

	Intact ACL	Intact ACL	Ruptured ACL	Ruptured ACL
	Medial meniscus	Lateral meniscus	Medial meniscus	Lateral meniscus
Grade 1	9	17	2	14
Grade 2	9	2	19	3
Grade 3	5	9	1	11
Grade 4	7	2	8	2

### Radiographic assessment

Radiographs were available for review in all 60 patients. OA was assessed in the radiographs separately for the medial tibiofemoral compartment, lateral tibiofemoral compartment and patellofemoral compartment. The results are shown in Tables 2 and 3.

**Table 2:** Osteoarthritis in patients with an intact ACL graft (*N* = 30)

Compartment	Grade 0	Grade 1	Grade 2	Grade 3	Grade 4
Patellofemoral	10	11	4	5	0
Medial	8	12	3	7	0
Lateral	10	12	4	3	1

**Table 3:** Osteoarthritis in patients with a ruptured ACL graft (*N* = 30)

	Grade 0	Grade 1	Grade 2	Grade 3	Grade 4
Patellofemoral	4	11	11	3	1
Medial	1	6	8	8	7
Lateral	7	12	6	1	4

Patients with ruptured ACL grafts showed significantly more OA of the medial tibiofemoral compartment than in those with an intact graft (*p* = 0.0003). OA of the lateral tibiofemoral compartment (n.s.) and patellofemoral compartment (n.s.) was found to be similar in patients with ruptured and intact ACL grafts.

In patients with ruptured ACL grafts, OA of the medial tibiofemoral compartment was more pronounced than that of the lateral tibiofemoral compartment (*p* = 0.013) as well as of the patellofemoral compartment (*p* = 0.002). OA was similar in the lateral tibiofemoral compartment and the patellofemoral compartment (n.s.).

No differences in terms of OA were shown between the different compartments in patients with an intact ACL graft when comparing medial versus lateral tibiofemoral compartments (n.s), medial tibiofemoral compartments versus patellofemoral compartments (n.s.), and patellofemoral versus lateral tibiofemoral compartments (n.s.).

In patients with an ACL graft rupture, there was an association between lateral meniscus injury and OA of the lateral tibiofemoral compartment (*p* = 0.001) and the patellofemoral compartment (*p* = 0.0021). There was also an association between injuries to the medial meniscus and OA of the patellofemoral compartment (*p* = 0.0099). There was no association between injuries to either the medial meniscus or the lateral meniscus and OA of the medial tibiofemoral compartment.

In patients with an intact ACL graft, there was no association between injuries to either the medial or lateral meniscus and OA.

### Graft tunnel in tibia

The location of the proximal tibia tunnel exit differed in patients with intact and ruptured ACL graft (*p* = 0.0470). In patients with an intact ACL graft, the tibia tunnel exit was in the anterior third for 17 patients and the middle third for 13 patients. In patients with a ruptured ACL graft, the tibia tunnel exit was in the anterior third for 25 patients and the middle third for five patients. None of the patients had a tibial tunnel exit in the rear third of the tibia.

### Knee joint laxity assessment

Results of anterior knee laxity using KT-1000 were available for 29 patients with an intact ACL graft and 30 patients with a ruptured ACL graft.

**Fig. 2:**
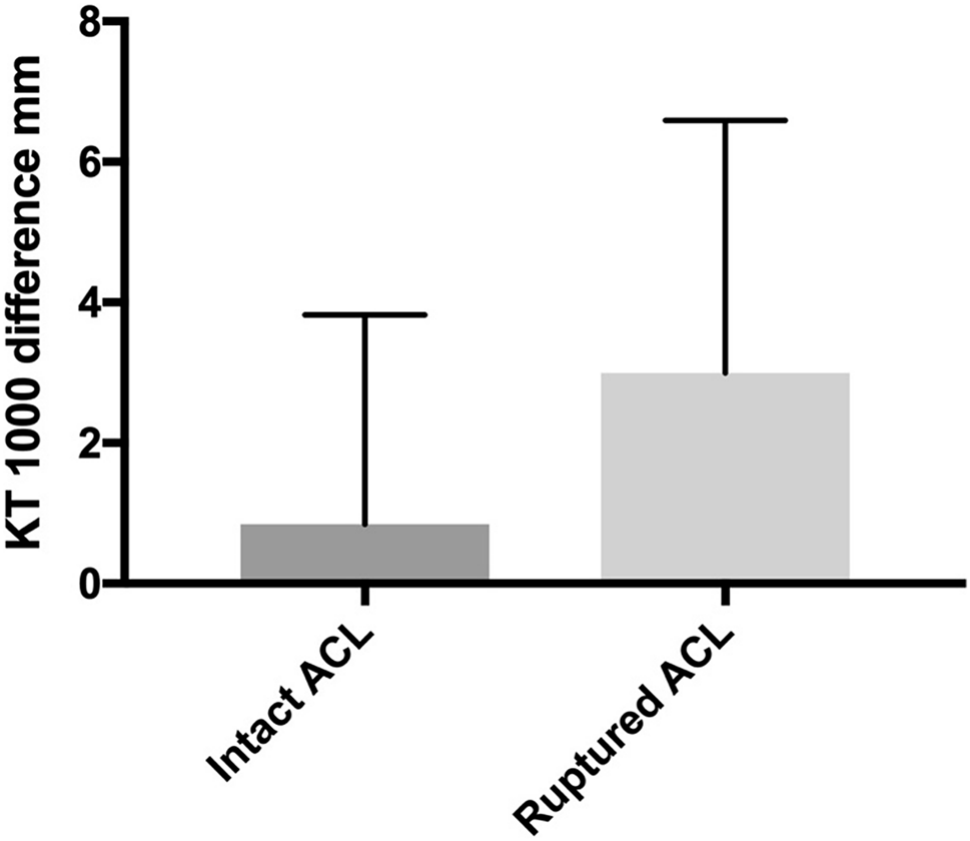
Side-to-side difference of anterior knee laxity measured with the KT-1000 in patients with an intact ACL graft, *N* = 29 and a ruptured ACL graft, *N* = 30

**Fig. 3:**
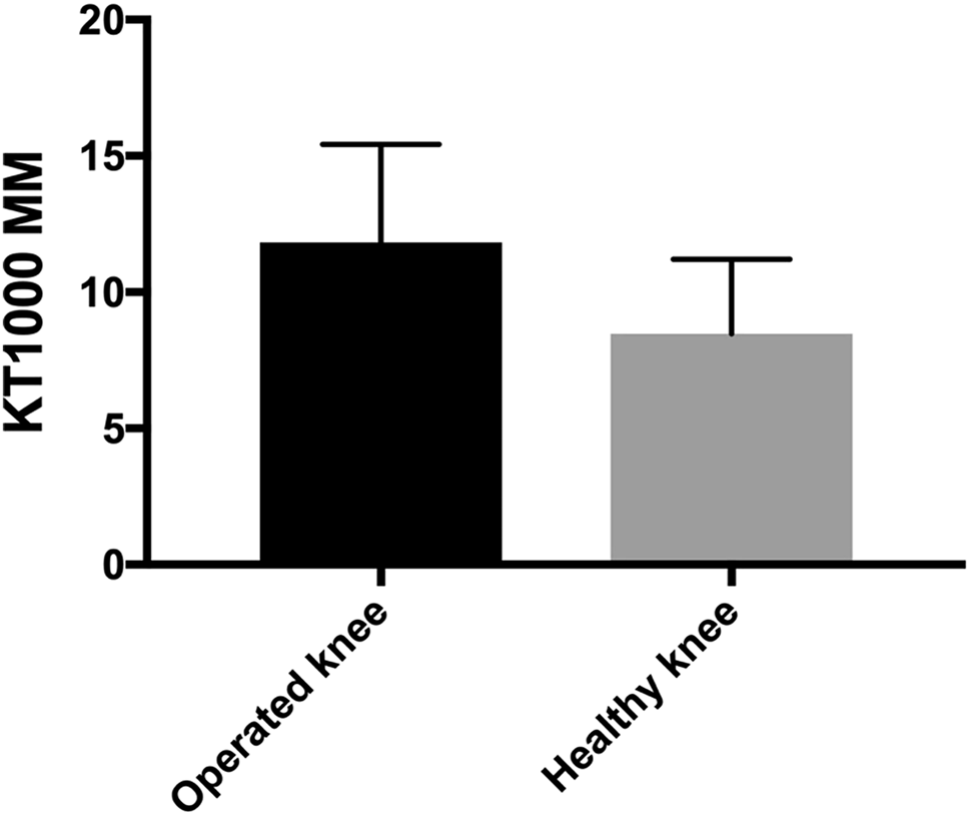
Anterior knee laxity of both knees with the KT-1000 in patients with a ruptured ACL graft, *N* = 30

Mean manual maximum side-to-side laxity difference was 0.8 mm ± 3 mm in patients with an intact ACL graft and 3 mm ± 3.6 mm in patients with a ruptured ACL graft. A side-to-side laxity difference of ≥ 3 mm was found in 16 patients with a ruptured ACL graft and in seven patients with an intact ACL graft. A larger anterior knee laxity difference was found in patients with a ruptured ACL graft than in those with an intact graft (*p* = 0.012) (Fig. 2). Side-to-side comparisons of anterior knee laxity in patients with a ruptured ACL graft showed higher KT-1000 results of the operated knee, mean 12 mm, than in the non-operated knee, mean 8 mm (*p* = 0.0003) (Fig. 3). In patients with an intact ACL graft, there was no side-to-side difference (n.s.).

**Fig. 4:**
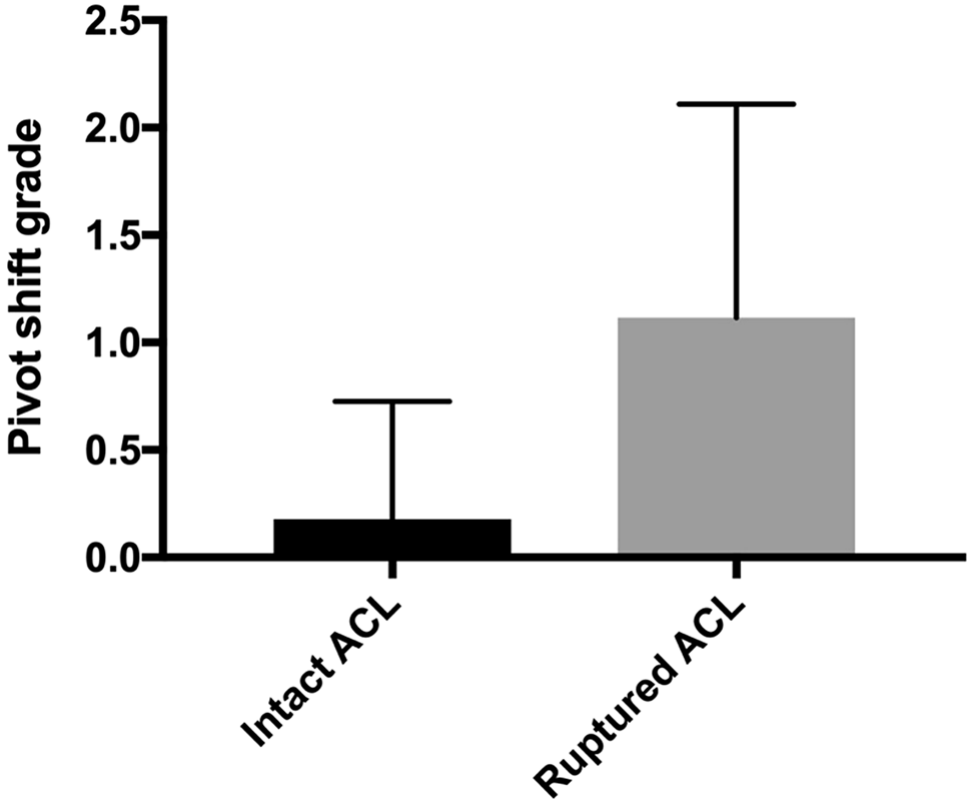
Knee joint laxity of both knees as evaluated with the pivot shift test in patients with an intact ACL graft, *N* = 28 and a ruptured ACL graft, *N* = 26

Knee joint laxity in the AP plane and internal rotation was examined in 28 patients with an intact ACL graft and 26 patients with a ruptured ACL graft (Table 4). It was not possible to provoke the pivot shift in six patients due to that these patients could not relax. Patients with a ruptured ACL graft were found to have a significantly higher grade pivot shift, than those with an intact graft (*p* < 0.0001) (Fig. 4).

**Table 4:** Knee joint stability evaluated with the pivot shift test (*N* = 54)

	Grade A (normal) 0	Grade B (nearly normal) 1	Grade C (abnormal) 2	Grade D (severely abnormal) 3	The test could not be performed
Pivot-shift test, patients with intact ACL graft, *N* = 28	25	1	2	0	2
Pivot-shift test, patients with ruptured ACL graft, *N* = 26	10	4	11	1	4

## Discussion

The most important finding of the present investigation was that the graft’s survival was 50% ≥ 30 years after ACL reconstruction and with a similar knee joint laxity in both knees. Furthermore, patients with ruptured ACL grafts showed more OA of the medial tibiofemoral compartment than those with an intact ACL graft.

A recent study estimated ACL graft survival to be 91% at 25 years following surgery [38], which is in contrast to the present findings of 50% intact ACL grafts at mean 31-year follow-up. In our previously published five to ten years results, only ten patients (11%) had clinical evidence of complete disruption of the ACL [20] whereas in the present study, 30 patients had ACL disruption (MRI). Many reasons have been suggested for graft failure after ACL reconstruction. The factors that increase the risk for ACL graft failure are not clearly understood but can be secondary to trauma, related to poor surgical technique, undiagnosed concurrent knee injuries, failed biologic incorporation of the graft [8], tunnel malposition [15], too early aggressive physical activity [8], young age [5], type of graft [8] and sex [40].

The Broström and Eriksson procedure has been proven to stabilize the knee joint in patients with chronic ACL insufficiency [29] and the surgeons who performed the ACL reconstructions in the present study were adequately trained. Poor surgical technique is therefore unlikely to explain the low graft survival.

The tibia and femoral tunnels can be classified as either anatomic, malpositioned or widened [15]. We found significantly more anteriorly placed tibia tunnels in patients with a ruptured ACL graft. This is in line with the findings of a previous report that a too anteriorly placed tibia tunnel can predispose for notch impingement, leading to graft attrition and failure [6]. Further, highly aggressive physical activity has been found to be a risk factor for ACL graft rupture [8] and there are also gender-related differences [40]. The gender distribution in the present study was very asymmetric with 92% men and merely 8% of women, which may reflect the situation in the 1960s. At that time, it was uncommon for women to participate in pivoting sports like football to the same extent as men. Most ACL injuries occur in football players [19]. Today, football is the sport that attracts the highest number of participants, especially in the age where most ACL injuries occur. This might explain the asymmetric gender distribution and the higher rate of graft ruptures in the present investigation. In contrast, there is a consensus in the literature that female athletes have a higher risk of sustaining an ACL injury than male athletes [36].

A significant increase in knee joint laxity was found in patients with a ruptured ACL, whereas those with an intact ACL graft showed a similar knee joint laxity of both their knees. The nearly normal knee laxity can partly be explained by the fact that many patients had an anterior tibia tunnel exit. It has earlier been shown that an increasingly anterior placement of the tibia tunnel results in significantly reduced anterior tibial translation as evaluated with the Lachman and pivot-shift tests [6]. In our previously published 5–10 years results of the present cohort, instrumented anterior knee laxity was reported excellent in 46 (69%) patients and fair or poor in 21 (31%) patients. In the present study, 23 (38%) patients had a side to side difference of 3 mm or more and 36 (61%) patients had a side to side difference of less than 3 mm. This reflects increased laxity over time, which can be explained by the increase of ACL ruptures between the follow-ups.

An ACL injury is often associated with meniscal and chondral injuries, which occur both at the time of the index trauma and secondarily over time in the ACL deficient knee [21]. Comparing the relative incidence of joint space narrowing before surgery, at the 5–10-year and the 28–33-year follow-up, there was a considerable increase in OA over time when both patients with an intact ACL graft and a ruptured ACL graft were added. In the present study patients with ruptured ACL grafts showed significantly more OA of the medial tibiofemoral compartment than those with an intact ACL graft. Furthermore, OA distribution was asymmetric in patients with ruptured ACL grafts. OA was most pronounced in the medial tibiofemoral compartment, in contrast to a previous [41] reporting a clear predisposition of OA in the lateral tibiofemoral compartment in patients with concomitant OA and ACL tear. The pronounced OA of the medial compartment could be a consequence of medial meniscus resection. This is in line with a previous report [22]. In the present cohort, the medial menisci were removed in 15 patients, eight in the group with ruptured ACL graft and seven in the group with an intact ACL graft. In the group with an intact ACL graft and medial meniscus resection, none of the patients had OA grade 4. In the group with a ruptured ACL graft and medial meniscus resection, there were seven patients with OA grade 4. This indicates that an intact ACL graft may play a role in terms of protection against OA of the medial tibiofemoral compartment in patients with a medial meniscus deficient knee.

Limitations of the present study were the retrospective design and that the patient cohort was heterogeneous, in the sense that some of the patients at the time of the index trauma already had experienced previous injuries to the knee (some requiring surgery) and were entering the study diagnosed with a collateral ligament injury, meniscal tears and/or OA. The exact extent of these additional lesions within the knee, besides damage to the ACL, was difficult to assess with certainty. Furthermore, MRI performed at that time utilized a low field strength magnet (0.2 T). However, one strength of the present study is that our patient material is unique and, to the best of our knowledge, this is the first study presenting data from a follow-up performed more than 30 years after ACL reconstruction. Moreover, the assessments were to a great extent unbiased in the sense that an orthopedic surgeon not involved in the ACL reconstruction, and from outside the faculty of our institution, performed the clinical tests (Lachman, pivot shift). Also, the same orthopedic surgeon had performed similar clinical tests of the present cohort in an earlier study performed five to ten years after the ACL reconstruction. The incorporation of objective measurements of anterior knee joint laxity adds to the impartiality of the results.

## Conclusion

Fifty percent of the patients showed an intact ACL graft and no side-to-side difference in terms of anterior knee laxity mean 31 years after ACL reconstruction. Patients with a ruptured ACL graft had more OA of the medial tibiofemoral compartment than those with an intact ACL graft.

## Abbreviations

ACL: Anterior cruciate ligament
OA: Osteoarthritis
MRI: Magnetic resonance imaging
IKDC: International Knee Documentation Committee
n.s.: Non significant
